# Reasons to move—a cross-sectional study to identify factors promoting regular exercise

**DOI:** 10.3389/fspor.2024.1515687

**Published:** 2024-12-11

**Authors:** Antonia Wambsganz, Katharina Köpl, Lukas Roell, Tim Fischer, Rebecca Schwaiger, Alkomiet Hasan, Andrea Schmitt, Peter Falkai, Isabel Maurus

**Affiliations:** ^1^ Department of Psychiatry and Psychotherapy, LMU University Hospital, LMU Munich, Munich, Germany; ^2^ Department of Psychiatry, Psychotherapy and Psychosomatics of the University Augsburg, Medical Faculty, University of Augsburg, Bezirkskrankenhaus Augsburg, Augsburg, Germany; ^3^ German Center for Mental Health (DZPG), Munich/Augsburg, Germany; ^4^ Laboratory of Neuroscience (LIM27), Institute of Psychiatry, University of São Paulo, São Paulo, Brazil; ^5^ Max Planck Institute of Psychiatry, Munich, Germany

**Keywords:** physical exercise, motivation, personality traits, self-efficacy, cross-sectional study, online survey, general population

## Abstract

Regular physical activity can prevent various physical and mental illnesses or improve their prognosis. However, only about half of the German population meets the WHO recommendations for physical activity. The aim of this study was to identify factors that influence engagement in regular exercise and could help increase physical activity levels in the general population. To this end, we conducted a cross-sectional study using questionnaire instruments and self-designed items. The research cohort comprised a sample of online-acquired data from 1,119 mentally healthy individuals. Higher regular exercise was associated with higher both intrinsic and extrinsic motivation, self-efficacy, resilience, internal locus of control, and risk-taking behaviour, as well as higher scores in the personality traits conscientiousness, extraversion, and agreeableness. Higher regular exercise was also linked to lower external locus of control. Whether participants exercised was also related to external circumstances, such as their financial situation, whether family members frequently exercised during childhood or the availability of sports facilities. Furthermore, participants' preferred exercise environment was found to be different from reality. Despite expressing a preference for outdoor and group exercise, most participants reported exercising alone and indoors. People who exercised regularly during childhood stated higher levels of intrinsic as well as extrinsic motivation and resilience. Based on our findings, we suggest that additional low-threshold, low-cost opportunities for physical exercise should be provided in public spaces that lack exercise facilities, as well as in childcare settings with a particular focus on disadvantaged social groups.

## 1 Introduction

Approximately 10% of all deaths in Europe are due to physical inactivity (1). This makes it one of the top 5 causes of death as it contributes to the development of cardiovascular, metabolic and musculoskeletal diseases (1). Physical inactivity also has a detrimental financial impact on health care systems worldwide, with an estimated cost of $ 47.6 billion per year between 2020 and 2030 ($502 billion in total over the decade) (2).

Therefore, the World Health Organization (WHO) recommends that adults engage in at least 150 min of moderate physical activity or 75 min of vigorous physical activity per week (3).

Regular physical activity has a wide range of benefits for both physical and mental health: On the one hand, it reduces the risk for myocardial infarction, cardiovascular disease in general, diabetes, and colorectal cancer (4, 5). On the other hand, it can be a preventative measure for common mental health conditions such as depression and anxiety and also has a beneficial effect on these conditions (6). Overall, mortality rates are reduced by up to 30% in people who engage in at least some physical activity compared to people who do not move at all (7, 8). However, according to national surveys, only 45%–51% of the adult population in Germany met these recommendations in 2019/2020 (9) and only 32% of the adult population in the European Union (10). Therefore, it is a desirable objective to increase the level of physical activity in the population, and hence reduce the morbidity and mortality.

Whether people ultimately avoid physical activity, engage in regular exercise, or even achieve peak performance as competitive athletes is largely attributed to the presence or absence of motivation in everyday life (11). Motivation can be described as the drive to achieve a goal that is perceived as valuable (12). Thus, if persons evaluate the target state of exercise positively, for example by recalling feelings of reward and stress relief, they are more likely to exercise to achieve said state. However, if the target state is evaluated negatively (e.g., due to sore muscles after training) the action is more likely to be avoided and, consequently, the individual may cease to exercise (11).

The self-determination theory proposes that an individual's basic needs include competence (self-efficacy), social relatedness, and autonomy (13). It further includes six so-called mini-theories that explain different aspects of motivation or personality functioning. These mini-theories suggest that the fulfilment of these basic needs has a positive influence on motivation, which in turn has a positive effect on physical activity. The mini-theories also differentiate between intrinsic and extrinsic motivation (13). Intrinsic motivation is present when an action is driven by a person's own will. In other words, when the means (action) and the ends (goal of action) are thematically congruent (14). Intrinsic motives may include both the performance of an activity (e.g., kinesthetic experiences) and the achievement of a result dependent on the activity (e.g., a new personal best) (14). In contrast, extrinsic motivation is present when an action is performed due to the anticipation of an aftereffect (e.g., money or recognition) (14). Meaning, the action is the result of an external influence (15, 16). Both intrinsic and extrinsic motives may coexist in any person (14). Previous studies conducted indicate a positive association between levels of intrinsic motivation and high physical activity (17, 18).

However, other factors such as personality traits and self-efficacy also seem to impact physical activity (19, 20). Furthermore, motivation for physical activity has been found to be influenced by various factors, such as a supportive environment or self-efficacy (11, 14). There is also some evidence to suggest that different personality traits may be linked to exercise behaviours (19) and also may impact motivation (21). Nevertheless, the current knowledge of the factors promoting and/or hindering physical activity is still scarce and incomplete.

There is a paucity of studies that have examined various factors such as motivation, personality traits, and in particular childhood sport history in the same group of subjects in a large-scale study population. Thus the aim of this study is to identify the principal factors that promote regular exercise.

To do this, we investigated the relationship between personality traits and intrinsic as well as extrinsic motives with the type and quantity of physical exercise engaged in. Higher intrinsic and extrinsic motivation was hypothesised to be associated with more frequent exercise. In line with self-determination theory, we also asked about self-efficacy, which corresponds to the basic need for competence; locus of control, which corresponds to the basic need for autonomy; and preference and actual setting for group exercise, which provides at least some insight into the fulfilment of the basic need for social relatedness.

Furthermore, this study explored associations between exercise behaviour during childhood and adolescence and the type as well as the amount of current physical activity. The hypothesis was that regular physical exercise and family support during childhood would be positively associated with regular physical exercise as an adult.

Additionally, this study evaluated facilitators and barriers to regular exercise and examined the impact of the resilience of study participants and their (sport-related) self-efficacy on the presence of physical exercise. It was anticipated that individuals with higher resilience and self-efficacy would be more likely to engage in physical exercise.

These findings can provide insights into how to improve conditions for regularly engaging in physical exercise and how to tailor interventions addressing related and relevant characteristics of respective participants.

## 2 Methods

### 2.1 Study design, population, and data extraction

Our study was approved by the local ethics committee of the Faculty of Medicine at the LMU Munich (registration number: 22-0625 KB) and complied with the Declaration of Helsinki.

This cross-sectional study was conducted in Germany using an anonymous questionnaire that could be completed online from any internet-enabled device via the online survey tool “SoSciSurvey” (22).

Inclusion criteria comprised being at least 18 years of age and prior informed consent regarding data collection. People with different levels of physical activity were surveyed, including professional athletes. There was no prior selection. Participants were recruited in a variety of ways, including emails to sports clubs and sport associations, clinics, students, and staff at the LMU Hospital Munich, social media (e.g., facebook groups for leisure swimmers), as well as well visibly placed flyers or posters on campus and in medical facilities. They had the chance to win one of 20 vouchers worth 20€ each. The recruitment period was between August 1st 2022 and December 18th 2022.

Upon completion of the survey, we categorised study participants into two groups according to whether they reported ever having been diagnosed with a mental illness or not. Separately, we identified a group of professional athletes who were members of a cadre with at least four training sessions per week and who participated in competitions such as world championships and Olympic Games. In this analysis, we focus on the sample of individuals who did not report a history of mental illness and were not professional athletes, i.e., the mentally healthy sample as detailed below.

### 2.2 Questionnaire

The questionnaire included standardised items on socio-demographic history and self-constructed items on physical and mental medical history and on detailed exercise history, both at present and during childhood. Data on body height and body weight were collected in order to calculate the body mass index (BMI). To further assess respondents' current weekly average of engaging in physical activity, the questionnaire also contained the Global Physical Activity Questionnaire (GPAQ) (23). Furthermore, the questionnaire inquired about barriers and facilitators for the implementation of physical exercise.

In addition, the following standardized instruments were implemented: The “Sport- und Bewegungsbezogene Selbstkonkordanz-Skala” (SSK scale) (24) surveyed different motives for exercising and the “SSA scale” (25) measured “self-efficacy for sporting activity”. To investigate the different motives of intrinsic and extrinsic motivation as important factors for physical activity according to the self-determination theory (13), we applied the “Exercise Motivations Inventory” (EMI-2; used in an abbreviated form) (26).

The Big Five Inventory 10 (BFI-10) (27) was used to survey the five dimensions of personality traits (extraversion, conscientiousness, agreeableness, neuroticism and openness). The Internal-External Control Belief Scale (IE-4) (28) was used to assess internal and external loci of control (the extent to which someone believes that the occurrence of an event is dependent on or independent of their own behaviour), and the General Self-Efficacy Short Scale (ASKU) (29) was used to assess general self-efficacy. Furthermore, the Brief Resilience Scale (BRS) (30) and the Brief Scale for Risk Taking (R1) (31) assessed resilience and risk affinity, while the Sport-Related Support Scale (32) evaluated support from friends and family. Reliability and validity information for the psychometric instruments used can be found in the Supplementary S1.

The questionnaire contained up to 190 questions and took about 20 min to complete. Depending on the information provided by the study participants, certain questions were automatically hidden or shown, providing further information on previous items (the full translated questionnaire can be found in Supplementary Table S2).

### 2.3 Statistical analyses

The descriptive preliminary analysis was carried out using Microsoft Excel version 16.73 (33). At first, we categorized the mentally healthy sample into an exercising and a non-exercising group. Said classification was based on the notion of whether respondents currently exercised regularly (at least once each week). This classification did not consider occupational activities or distances travelled by foot or by bicycle that were not undertaken for the purpose of physical exercise. Similarly, we classified participants depending on whether they engaged in regular exercise during childhood or not, irrespective of physical education classes in school.

IBM SPSS version 29.0.0.0 (34) was used for further analysis and to characterise our sample by frequencies, means, medians and standard deviations. It was also used to calculate the participants' body-mass-index (BMI) as ratio of body weight in kilograms to height squared in meters.

To compare two different groups - for example, those who exercise and those who do not - we used independent *t*-tests for metric data, Cohen's d to measure the effect size of significant results and the Welch method to correct for unequal variances. Mann-Whitney *U*-tests and Chi-Square-tests were used for non-metric data. We also used binary logistic regression to examine the influence of a personality trait on the likelihood of regular exercise or to examine the influence of social support on the likelihood of regular exercise. Pearson correlations were performed for metric data and Spearman correlations for non-metric data. A distinct quantity of responses (*n*) was available for each question, which can be identified by the degrees of freedom (df).

## 3 Results

### 3.1 Demographic characteristics

1,746 people gave their informed consent and started to participate in the survey, of whom 1,577 responded to at least the sociodemographic and somatic history questions and were therefore included in our analysis. 1,376 participants completed the entire questionnaire. This publication focuses on the 1,119 individuals without a known history of mental illness, here referred to as the mentally healthy sample. The remaining 458 participants were either professional athletes or reported having been diagnosed with a mental illness.

The mean age in our mentally healthy sample was 38.3 ± 15.5 years, 66.5% (*n* = 744) were female and 33.5% (*n* = 375) male. Most respondents had the highest German school degree: 68.0% (*n* = 761). Financial worries were reported by 7.1% (*n* = 80) of the mentally healthy sample. A detailed characterisation of participants can be found in Supplementary Table S3.

### 3.2 Determining factors of exercise behaviour

Overall, 73.1% (*n* = 754) of the mentally healthy sample reported to engage in regular exercise at the time of the study, 70.2% of the female participants and 78.6% of the male participants. Most exercised one to three times a week (58.3%), 29.3% exercised 4–5 times and 12.4% exercised six times or more. For details on the participants' levels of physical activity as assessed with the GPAQ (23) see Table 1. In our exercising group, 92.7% met the WHO recommendations of taking part in at least 150 min of physical activity each week, 89.9% through leisure activities alone. In comparison, in our non-exercising group, only 51.8% met the WHO recommendations. Overall, the WHO recommendations were met by 81.8% of our mentally healthy sample. See Table 1 for more information about the participants' daily physical activity levels. Those who exercised regularly reported a slightly lower Body-Mass-Index (BMI) than our non-exercising group (23.31 vs. 24.55 kg/m^2^; *p* < 0.001, *t*(393) = 3.94, MD = 1.24, Cohen's *d* = 3.92, 95% CI [0.18, 0.46]).

**Table 1:** Daily physical activity levels as assessed by the GPAQ.

	Descriptive statistics - mean (SD)			*t*-test: comparison between exercising sample and non-exercising sample					
	Total mentally healthy sample (*n* = 1,119)	Exercising sample (*n* = 754)	Non-exercising sample (*n* = 278)	*p*	df	T	MD	Cohen's d	95% CI
Daily moderate leisure activity in minutes	19.1 (25.8)	23.7 (27.5)	12.1 (20.4)	<0.001	665	−7.33	−11.58	25.81	[−0.59, −0.31]
Daily intensive leisure activity in minutes	22.1 (34.0)	30.7 (37.6)	4.8 (12.4)	<0.001	1,021	−16.61	−25.90	32.80	[−0.93, −0.65]
Daily moderate work activity in minutes	19.2 (51.9)	19.6 (51.7)	22.5 (57.0)	0.224					
Daily intensive work activity in minutes	7.8 (43.1)	5.8 (27.8)	8.1 (43.8)	0.207					
Daily total activity in minutes	68.2 (97.3)	79.9 (92.8)	47.5 (99.0)	<0.001	1,030	−4.88	−32.35	94.53	[−0.48, −0.20]
Daily transport activities in minutes	23.9 (29.6)	25.8 (29.1)	25.2 (32.6)	0.415					
Daily sitting in minutes	457.3 (649.6)	492.1 (724.7)	510.9 (454.6)	0.344					

Table 1 presents descriptive statistics for the GPAQ for the total mentally healthy sample, the exercising sample and the non-exercising sample and a comparison of means using an independent *t*-test between the exercising and non-exercising group. GPAQ, Global physical activity questionnaire (23), SD, standard deviation.

The mean age in the exercising group was 39.01 years (SD = 15.99) and 37.34 years (SD = 14.33) in the non-exercising group. There was no significant correlation between age and exercise behaviour nor did exercise behaviour differ based on participants' income. However, we found a significant difference between the two groups regarding the subjectively reported variable “health status” (U = 84,260.5, Z = −4.79, *p* < 0.001) as well as for the variable “highest educational attainment” (U = 97,292.5, Z = −2.00, *p* = 0.045). Both variables showed higher values in the exercising group.

**Figure 1:**
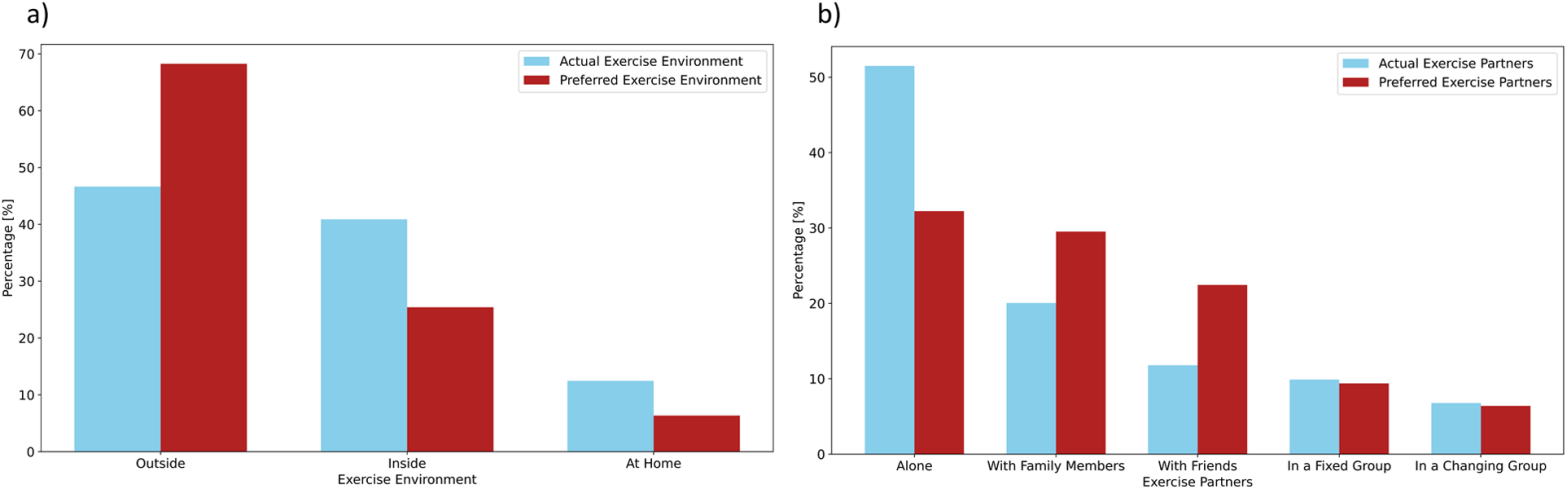
Exercise setting in the mentally healthy sample. **(a)** compares the actual and preferred exercise setting in the mentally healthy sample (*n* = 741–746) about location and **(b)** with regard to companionship.

As illustrated in Figure 1, most of the mentally healthy sample (68.3%) reported a preference for exercising outdoors, whereas only 46.6% actually do so. Similarly, indoor, and at-home sports are practised much more frequently than theoretically preferred. There are also clear differences between preferred and actual levels of companionship. Although more than half of the mentally healthy sample (51.5%, *n* = 383) reported mostly exercising alone, the majority actually expressed a preference to exercise with friends [32.2% (*n* = 239)] or in a group [28.8% (*n* = 214)], whereas only 29.6% (*n* = 220) favoured exercising alone. Overall and noteworthy, discrepancies between preferred and actual settings were significant.

#### 3.2.1 Personality traits in the mentally healthy sample

We further investigated whether the personality traits extraversion, conscientiousness, agreeableness, neuroticism, and openness as evaluated with the BFI-10 (27) were associated with the participants' exercise behaviour as well as preferred setting and kind of exercise.

**Figure 2:**
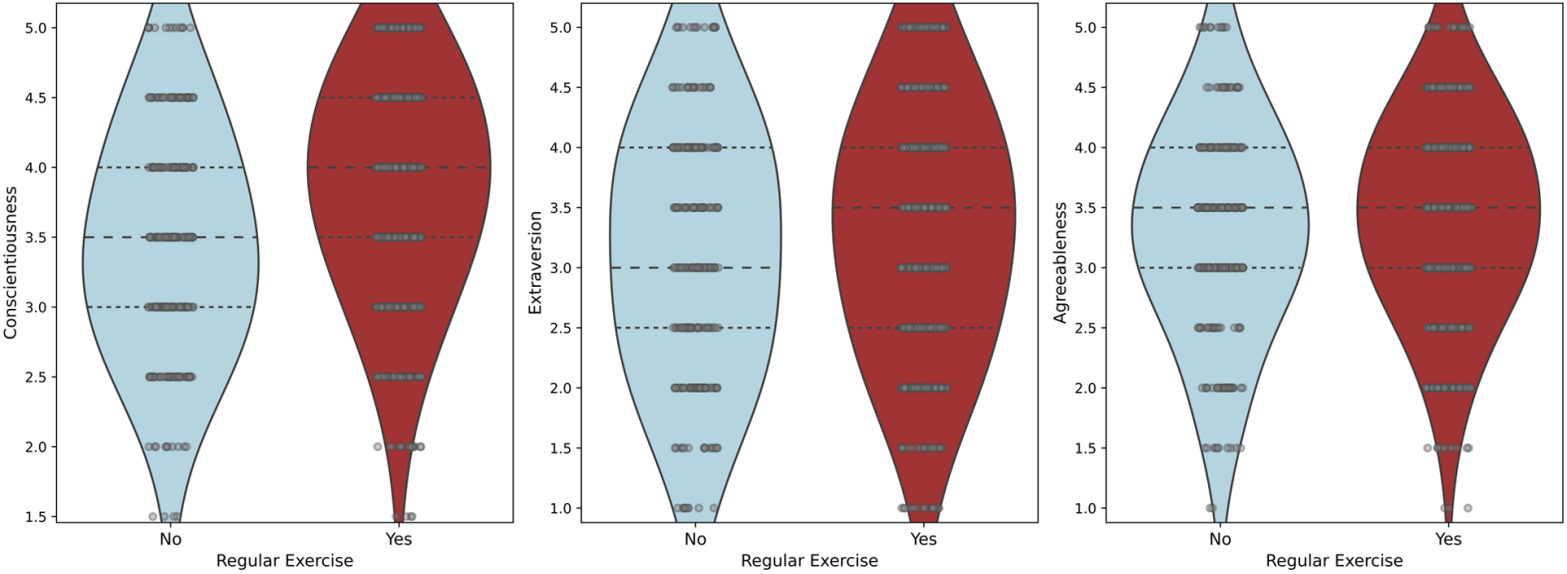
A Comparison of the non-exercising and the exercising group on conscientiousness, extraversion and agreeableness scores is presented using violin plots.

**Figure 3:**
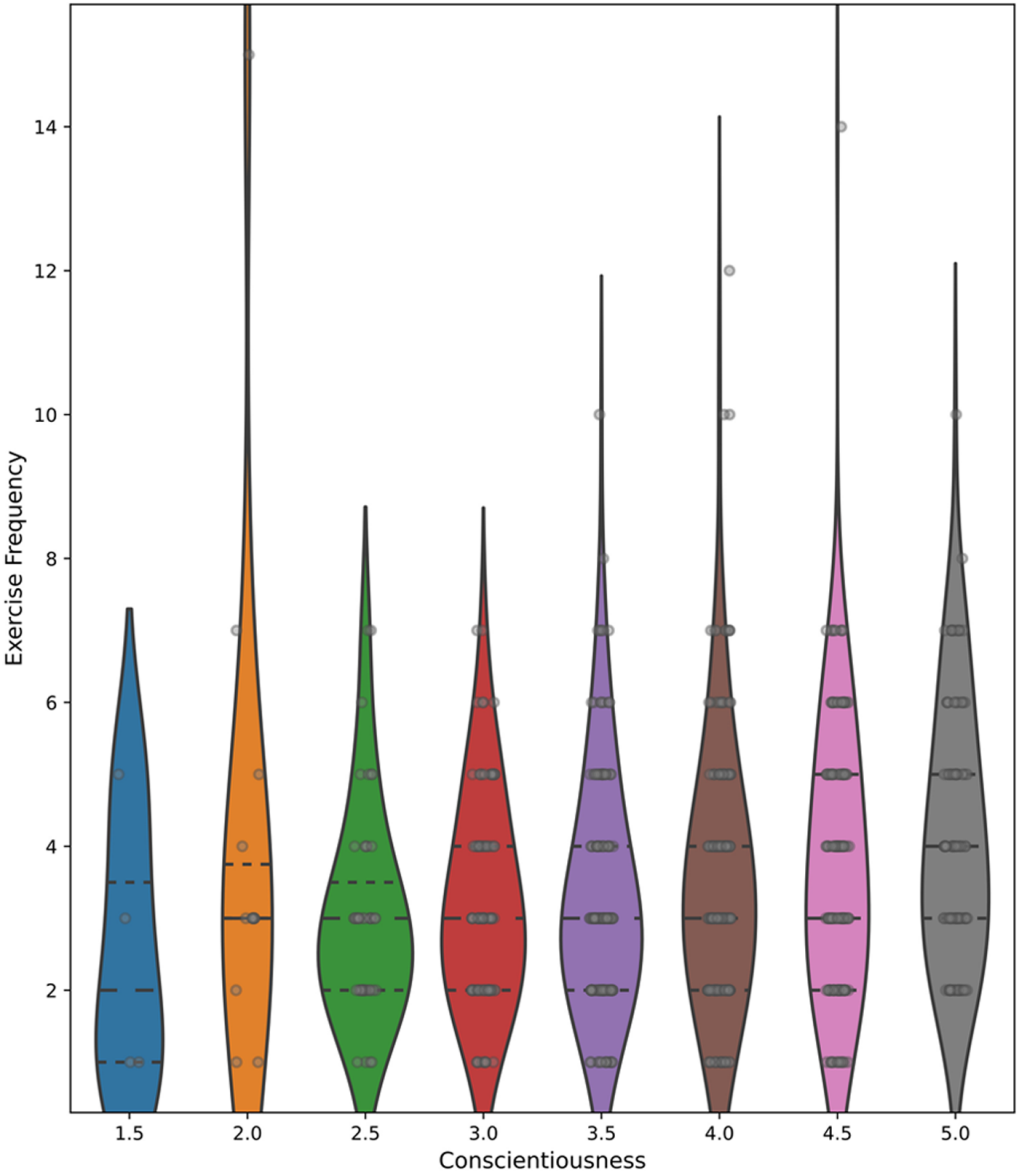
Relationship between exercise frequency and conscientiousness score in the exercising group.

Independent *t*-tests and binary logistic regressions showed that the personality trait conscientiousness (*p* < 0.001, *t*(998) = −7.55, MD = −0.42, Cohen's *d* = 0.78, 95% CI [−0.68, −0.40]) (OR = 0.95), as well as the traits extraversion (*p* = 0.022, *t*(998) = −2.29, MD = 0.17, Cohen's *d* = 1.01, 95% CI [−0.30, −0.02]) (OR = 0.17), and agreeableness (*p* = 0.022, *t*(997) = −2.30, MD = −0.131, Cohen's *d* = 0.802, 95% CI [−0.30, −0.02]) (OR = 0.22) were significantly related to whether or not a participant engaged in regular exercise (see Figure 2 for violin plots). Namely, participants who exhibited a stronger expression of these traits were more likely to engage in regular exercise. However, there was no significant association between the personality traits neuroticism or openness and exercise behaviour. Within the group of regular exercisers, higher levels of conscientiousness correlated positively with exercise frequency (*r* = 0.16, *p* < 0.001, *n* = 729) (see Figure 3 for violin plots).

In terms of exercise setting, people who exercised with others rather than alone had higher levels of agreeableness (*p* = 0.046, *t*(723) = −2.00, MD = −0.12, Cohen's *d* = 0.78, 95% CI [−0.29, −0.002]). No notable distinctions in other personality traits were observed between those groups.

We also found no discernible correlations between personality traits and the preferred kind of sports.

In conclusion, we observed that regular exercise was most closely linked to the personality traits extraversion, conscientiousness, and agreeableness. Conscientiousness was also significantly related to the frequency of exercise and agreeableness to the setting of exercise.

#### 3.2.2 Self-efficacy, locus of control, resilience and risk taking

**Figure 4:**
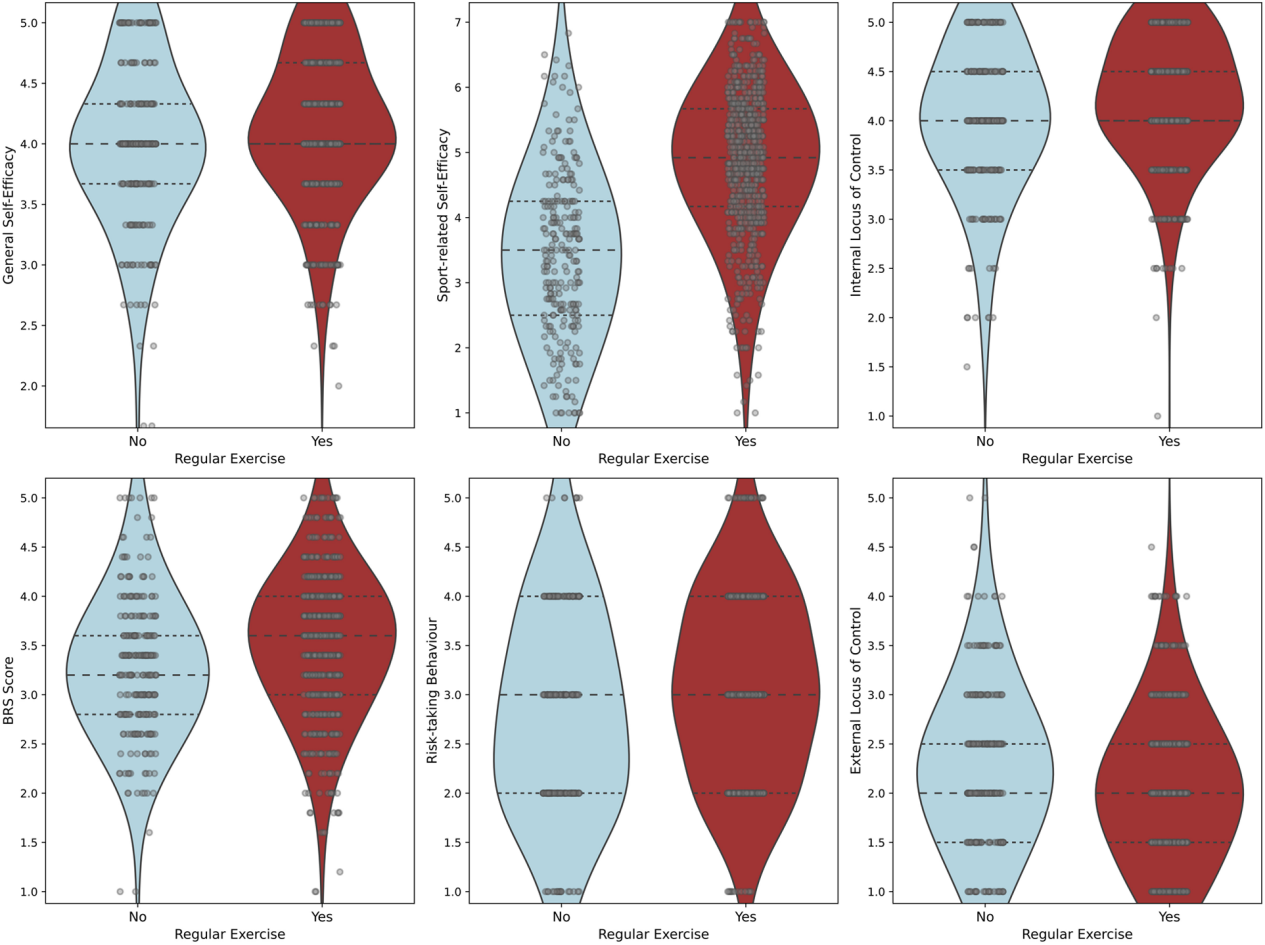
Relationship between self-efficacy, locus of control, resilience and risk taking and whether someone exercises regularly. The violin plots compare the general and sport-related self-efficacy, the internal and external locus of control, the resilience and the risk-taking behaviour scores for the non-exercising and the exercising group.

We also evaluated if engaging in regular exercise was associated with increased levels of self-efficacy, an internal locus of control, and resilience scores. Our results showed that the exercise group had significantly higher ratings in both general self-efficacy (ASKU score) (*p* = 0.002, *t*(995) = −3.09, MD = −0.13, Cohen's *d* = 0.59, 95% CI [−0.36, −0.08]) and sport-related self-efficacy (SSA score) (*p* < 0.001, *t*(402) = −15.37, MD = −1.41, Cohen's *d* = 1.17, 95% CI [−1.35, −1.05]), a rather internal locus of control (IE-4 score) (*p* < 0.001, *t*(996) = −4.24, MD = −0.193, Cohen's *d* = 0.64, 95% CI [−0.44, −0.16]) as well as higher scores in resilience ratings (BRS score) (*p* < 0.001, *t*(993) = −4.54, MD = −0.223, Cohen's *d* = 0.69, 95% CI [−0.46, −0.18]) when compared to the non-exercising group. In addition, the exercise group reported higher scores for risk taking (R1 score) (*p* < 0.001, *t*(428) = −3.64, MD = −0.25, Cohen's *d* = 0.91, 95% CI [−0.42, −0.14]). By contrast, an external locus of control was more pronounced in the non-exercising group (IE-4 score) (*p* < 0.001, *t*(447) = 3.70, MD = 0.21, Cohen's *d* = 0.76, 95% CI [0.13, 0.41]). The corresponding violin plots are shown in Figure 4.

#### 3.2.3 Intrinsic and extrinsic motivation

As hypothesised, we found an association between intrinsic and extrinsic motivation as assessed by the EMI-2 (26) and exercise behaviour in the mentally healthy sample. Both intrinsic and extrinsic motivation were significantly related (*p* < 0.001) to whether a person engages in physical exercise or not (intrinsic: *t*(351) = −13.93, MD = −1.10, Cohen's *d* = 0.93, 95% CI [−1.34, −1.04]; extrinsic: *t*(410) = −7.20, MD = −0.46, Cohen's *d* = 0.82, 95% CI [−0.70, −0.42]). On average, motives related to intrinsic motivation were upwards of 20% more pronounced in the exercising than in the non-exercising group (on a scale of 0–5) (*t*-tests with *p* < 0.001): enjoyment (*t*(391) = −16.61, MD = −1.73, Cohen's *d* = 1.30, 95% CI [−1.48, −1.18]), challenge (*t*(998) = −12.72, MD = −1.34, Cohen's *d* = 1.48, 95% CI [−1.05, −0.76]), revitalisation (*t*(359) = −12.54, MD = −1.21, Cohen's *d* = 1.13, 95% CI [−1.22, −0.92]), stress management (*t*(381) = −11.18, MD = 1.20, Cohen's *d* = 1.31, 95% CI [−1.06, −0.77]). Moreover, the extrinsic motive affiliation was also higher in the exercising group (*t*(525) = −8.67, MD = −1.05, Cohen's *d* = 1.77, 95% CI [−0.73, −0.45]). The remaining internal (competition, nimbleness, positive health, strength and endurance) and external motives (appearance, ill health avoidance, positive health, social recognition) were between 10% and 20% higher for those who exercised. We found no statistically significant differences for the extrinsic motives of social pressure and weight management.

In conclusion, the most important motives in our study appear to be enjoyment, challenge, revitalisation, stress management and affiliation. Intrinsic motivation seems to be more important than extrinsic motivation when it comes to regular physical exercise.

**Figure 5:**
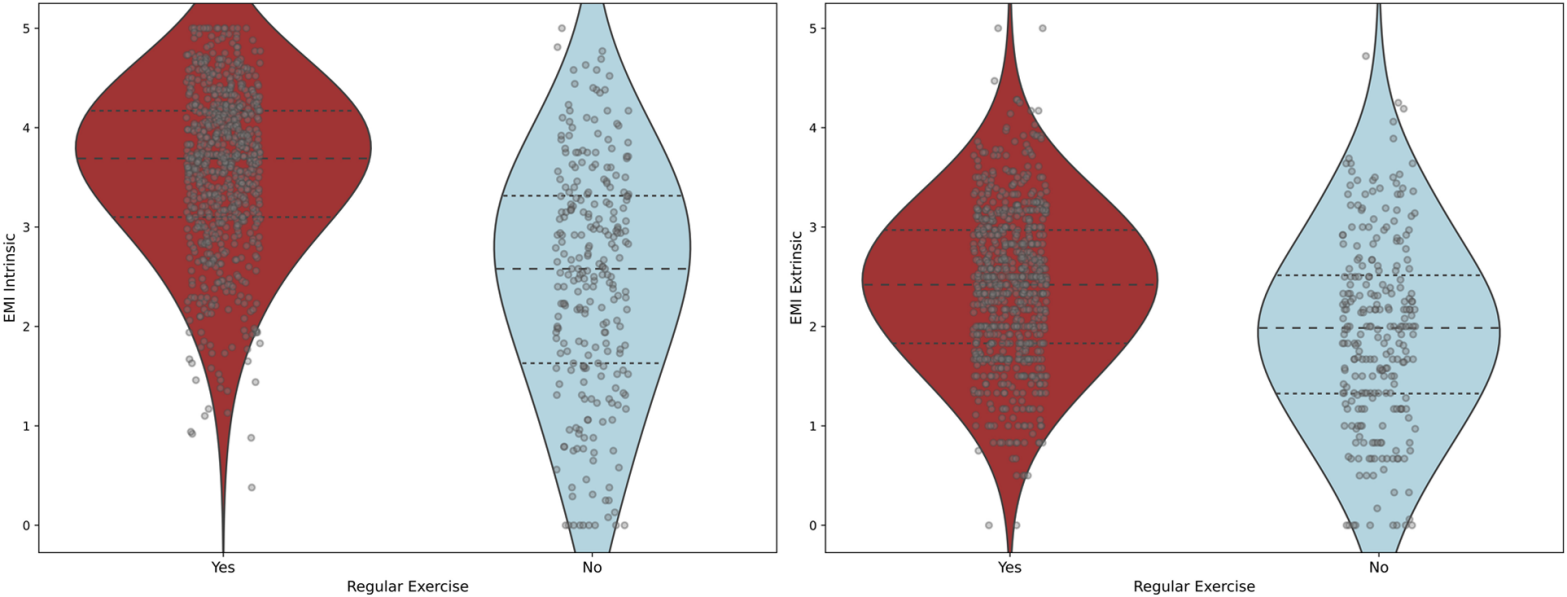
Comparison of the exercising and the non-exercising group on intrinsic and extrinsic motivation.

A detailed summary of the EMI-2 results can be found in Supplementary Table S4, corresponding violin plots in Figure 5.

#### 3.2.4 Social support

Contrary to our assumption, in our mentally healthy sample we found no significant relationship between experiencing exercise-related family support during childhood and the likelihood of engaging in regular exercise in the present. Among those in the mentally healthy sample who exercised in childhood, 70.1% had at least one other exercising family member. In comparison, only 27.1% of the inactive childhood group had an exercising family member.

Thus, it appears that family support itself during childhood does not directly influence an individual's likelihood of exercising regularly, whereas the presence of other exercising family members does.

#### 3.2.5 Facilitators and barriers to regular exercise

Examining the participants' reported barriers and facilitators to regular exercise (or even more exercise in the case of the exercising sample), the main barriers for the physically inactive group were lack of time due to work and a lack of drive and energy (see Table 2). Both groups reported that a short distance to the training venue and fixed appointments were reported to be the most helpful (see Table 3).

**Table 2:** Barriers to regular exercise.

	Non-exercising sample (*n* = 252–267)		Exercising sample (*n* = 693–718)		Mann-Whitney-*U*-test			
	M	SD	M	SD	*p*	U	Z	*r*
Financial barriers	1.89	1.13	1.70	1.00	0.02	86,446.00	−2.32	−0.07
Lack of knowledge	2.46	1.26	1.59	0.87	<0.001	56,961.00	−10.60	−0.34
Missing offer	2.00	1.14	1.61	1.00	<0.001	74,921.50	−5.65	−0.18
Unaware	1.77	1.08	1.28	0.68	<0.001	71,152.00	−7.97	−0.26
No time due to family	2.29	1.36	1.82	1.07	<0.001	77,861.50	−4.80	−0.15
No time due to work	3.14	1.44	2.53	1.32	<0.001	71,394.50	−6.07	−0.19
No time due to other matters	2.50	1.22	1.83	1.01	<0.001	59,878.50	−7.86	−0.26
Too unathletic	2.35	1.28	1.48	0.81	<0.001	56,405.00	−10.93	−0.35
Shame	2.04	1.27	1.44	0.82	<0.001	70,964.00	−7.42	−0.24
Fear of embarrassment	2.09	1.28	1.52	0.88	<0.001	71,851.00	−6.86	−0.22
Concern for injury	1.75	0.98	1.65	0.92	0.125			
Lack of drive and energy	3.38	1.24	2.03	1.04	<0.001	40,640.00	−14.24	−0.45
Pain or discomfort during exercise	1.94	1.19	1.52	0.81	<0.001	78,191.50	−4.95	−0.16
Disappointed by previous experiences	1.95	1.16	1.50	0.83	<0.001	74,177.50	−6.06	−0.19
Bigger worries in life at the moment	2.59	1.38	1.74	0.99	<0.001	61,141.50	−9.11	−0.29

Table 2 shows the barriers to regular (or even more) physical exercise in both the exercising sample (*n* = 693–718) and the non-exercising sample (*n* = 252–267). Participants were requested to assess, on a scale of 1 (does not apply at all) to 5 (fully applies), the degree to which each item listed impeded their exercise behaviour. M, mean; SD, standard deviation.

**Table 3:** Facilitators to regular exercise.

	Non-exercising sample (*n* = 256–263)		Exercising sample (*n* = 697–717)		Mann-Whitney-*U*-test			
	M	SD	M	SD	*p*	U	Z	*r*
Further education	2.06	1.12	1.85	1.09	0.002	80,645.00	−3.06	−0.08
Fixed appointments	3.44	1.31	3.42	1.38	0.92			
Flexible training in terms of time and place	3.10	1.27	3.06	1.37	0.71			
Reminder by phone or email	1.94	1.13	1.64	0.99	<0.001	75,475.50	−4.29	−0.13
Short distance to the training location	3.73	1.25	3.83	1.29	0.12			
Trainings free of charge	3.38	1.41	3.26	1.47	0.35			
Group with equally fit people	3.24	1.38	3.13	1.39	0.25			
Coaches with knowledge of physical conditions	2.53	1.43	2.40	1.43	0.19			

Table 3 shows the facilitators to regular (or even more) physical exercise in both the exercising sample (*n* = 697–717) and the non-exercising sample (*n* = 256–263). Participants were asked to assess, on a scale of 1 (does not apply at all) to 5 (fully applies), the extent to which each of the items listed would be helpful in order to increase their amount of physical exercise. M, mean; SD, standard deviation.

Of note, study participants with lower scores on the personality trait of conscientiousness gave higher scores for the facilitator “reminders by phone or email” (*r* = −0.18, *p* < 0.001, *n* = 957).

#### 3.2.6 Physical exercise during childhood

To evaluate the impact of exercise during childhood on present outcomes, we additionally gathered data on exercise-related characteristics in childhood. 78% of the mentally healthy sample reported having exercised regularly during childhood (76.9% in women and 80.1% in men). Approximately 80% of individuals who participated in regular exercise during their respective childhoods were affiliated with sports clubs, with a 78.2% membership percentage below the age of 12 and 81.3% above the age of 12. Interestingly, individuals who exercised regularly in childhood were more likely to maintain their respective exercise habits in adulthood. 76.8% of those who exercised regularly during childhood still exercise regularly today, in contrast to only 60.1% in the comparison group [x^2^ (1) = 24.39, *p* < 0.001].

In our study, people who regularly exercised during childhood reported higher levels of education (U = 90,449.0, Z = −3.52, *p* < 0.001) and income (U = 90,882.5, Z = −2.93, *p* < 0.001) compared to those who did not. However, we found no difference in subjectively reported current health status or BMI.

##### 3.2.6.1 Relationship between exercise behaviour during childhood and personality characteristics

Individuals who reported regularly engaging in exercise as children scored higher on extraversion (*p* < 0.001, *t*(998) = −3.54, MD = −0.27, Cohen's *d* = 1.01, 95% CI [−0.42, −0.12]) and lower on neuroticism (*p* < 0.001, *t*(998) = 3.94, MD = 0.28, Cohen's *d* = 0.94, 95% CI [0.15, 0.45]) than individuals who did not exercise regularly in childhood. Moreover, they showed significantly higher levels of both intrinsic and extrinsic motivation towards exercise (*p* < 0.001, *t*(305) = −7.65 and *t*(987) = −4.40, MD = −0.28 and −0.67; Cohen's *d* = 1.01 and 0.84, 95% CI [−0.81, −0.51] and [−0.49, −0.19]). In a further comparison of the two groups, we also found significantly higher values of internal locus of control, resilience, self-efficacy and risk-taking for those who engaged in regular exercise during childhood. However, the personality traits agreeableness, conscientiousness, and openness, as well as external locus of control showed no significant difference.

##### 3.2.6.2 Discontinuation of sport in childhood

More than two-thirds (69.0%) of participants from the mentally healthy sample reported cessation of participation in a sport during childhood/adolescence. The most common reason given by 42.0% (multiple choice) was “other hobbies were more important”. The second most common reason was “no fun”, given by 36.0%. The reasons “didn't get on with coach”, “too much pressure to succeed” and “financial reasons” were selected by 8.6%, 6.2%, and 5.3% of participants, respectively.

Over 43.4% of study participants expressed a desire to engage in a certain sport during childhood but the inability to do so. The most common reason for this was “no facilities nearby” with 42.3%, followed by “no family support” with 33.1%, and “financial reasons” with 26.9%.

## 4 Discussion

This study aimed to identify determinants of exercise behaviour to contribute to a better understanding of how to promote optimal conditions for regular physical activity in the general population. The majority of participants in our study exercised regularly and met the WHO criteria of at least 150 min of moderate physical activity or 75 min of vigorous physical activity per week. While most of the participants reported a preference to exercise outdoors with friends or in groups, the number of individuals actually doing so was significantly lower. While there was no significant correlation between age and exercising behaviour, the presence of different motives and individual personality traits were linked to engaging in regular exercise. Specifically, we observed significant positive associations between exercise behaviour and the personality traits conscientiousness, extraversion, and agreeableness and between exercise behaviour and both intrinsic and extrinsic levels of motivation, with intrinsic motives appearing to play a more important role. In addition, levels of self-efficacy, resilience, internal locus of control and risk taking showed positive associations with exercise behaviour. However, a significant negative association was found for an external locus of control.

Moreover, people who exercised regularly during childhood had a notably higher proportion of active family members compared to those who didn't exercise as children. Lastly, people who exercised regularly as children were more likely to exercise regularly as adults.

According to previous studies, only about half of the adult population met the WHO recommendations (1, 9), whereas in our sample about 4 out of 5 people did. This might be due to our recruitment being biased towards individuals with a sports-related background or because the topic might have appealed more to sports enthusiasts, resulting in a higher proportion of physically active participants in our study.

Our findings on the relationship between personality characteristics and exercise behaviour are in line with previous literature and said to be independent of factors such as gender, age or culture/country (35, 36). Individuals with high levels of conscientiousness are regarded as disciplined, self-regulated, dutiful, and deliberate (37), which favours the initiation and maintenance of physical exercise. Similarly, people with higher levels of extraversion are more prone to seek out social contact and sensory stimulation (37), which can be a part of engaging in exercise. Those with a higher level of agreeableness are considered to be compassionate, humble, trusting, cooperative, and altruistic (37) which can also be beneficial for maintaining physical exercise. Whereas the positive correlations between physical exercise and the personality traits extraversion, conscientiousness and agreeableness are in line with a recent cross-sectional study conducted in 4,244 German students and with previous meta-analyses (19, 35, 36), our study could not confirm a positive association between physical exercise and the personality trait openness as well as a negative association with neuroticism that has been previously suggested (19, 36). This discrepancy may be attributed to the exclusion of individuals diagnosed with a mental illness, a group known to exhibit elevated neuroticism scores (38). Said results underscore the necessity to tailor exercise regimens to individual needs and personality traits, such as, for example, cues and/or reminders via telephone for people low in conscientiousness. For individuals with low levels of extraversion and/or agreeableness, participation in open settings, such as open days or direct contact in public spaces may be beneficial.

Our finding of a positive association between intrinsic and extrinsic motivation and regular exercise behaviour is consistent with previous research (39). The motives rated as most significant in our exercising sample – enjoyment, stress management, challenge, revitalisation and affiliation – are also among the most commonly reported motives in other studies (40, 41). If prevention is to be further strengthened in the German health care system, it would be important to start with these motives. The keywords enjoyment, stress management, challenge, revitalisation and affiliation can be used to draw attention to exercise programs, promote workplace exercise and target health insurance members. Exercise regimens could be planned in cooperation with those concerned, thereby increasing intention to exercise. Previous studies have shown that motivation can be influenced by using behaviour change techniques. These behaviour change techniques may include, for example, behavioural goal setting, action planning, behavioural feedback, behavioural instruction, or behavioural demonstration (42).

Other studies also found a positive relationship between self-efficacy and exercise behaviour (43). Intervention studies have used a variety of methods to increase self-efficacy levels, such as face-to-face and telephone counselling, email feedback, discussion groups or behaviour change classes. They have shown small but significant effects on self-efficacy and subsequently on physical activity levels, focusing mainly on lifestyle physical activity such as walking and gardening (43). Future research should build on this to identify further effective interventions to increase self-efficacy and, therefore, exercise and physical activity in the general population.

In our study, people who exercise regularly show increased self-efficacy, a stronger internal locus of control and higher overall and intrinsic motivation compared to non-exercisers. In addition, the majority of our study population prefer to exercise with others rather than alone. This is consistent with the self-determination theory (13).

Regarding additional barriers and facilitators of regular exercise, many participants indicated that proximity as well as flexible training times and locations would be helpful. Correspondingly, the most common reason given for not exercising regularly during childhood was a lack of sports facilities in the area. To address this, more low-threshold public exercise opportunities should be provided in public areas and where few sports facilities exist. This could be achieved by the establishment of weekly exercise groups at different levels that can be joined without prior registration and free of charge. The construction and maintenance of public fitness facilities like outdoor gyms, safe and accessible cycle paths and public parks could also animate people and encourage them to spend more time outdoors exercising. Accordingly, a study conducted in Chile found that the presence of more outdoor gyms increases the physical activity in the population and increases the likelihood of meeting the WHO recommendations for physical activity (44).

Low-threshold and low-cost or no-cost opportunities in childcare facilities should also be created and promoted, especially for those from socially disadvantaged backgrounds, as these children generally have less opportunity to engage in physical activity (45). Our data supports this notion as many individuals reported not engaging in or quitting their preferred sport due to a lack of financial resources. A Brazilian study found that the majority of outdoor gym users were people with low income, so the installation of outdoor gyms could reduce social inequalities in physical exercise (46).

Another approach would be to introduce and promote more free, well-developed fitness apps, supported, for example, by health insurance bonus programs. This would have the advantage of being flexible in terms of place and time, but the disadvantage of losing the social aspect of exercise, which also seems to play an important role, as most of our study participants stated that they prefer to exercise with others rather than alone.

Our data also shows a significant association between the exercise behaviour of family members and respondents during their respective childhoods. Moreover, engaging in regular exercise during childhood seems to make regular exercise more likely in the present. Thus, the exercise behaviour of family members appears to have a significant and lasting influence on exercise levels of people in child- and adulthood. In conclusion, exercise interventions, particularly those targeting family members could be beneficial to increasing physical activity levels in the population. Establishing programs parents and children attend together could be a possible solution to said problem.

When interpreting our findings, several limitations have to be considered. Although the online format of our survey allowed us to reach a large number of participants from different backgrounds, there are constraints within this format. As our survey was predominantly advertised in sporting environments and at universities, our results are not representative of the wider population. It is possible that a sampling bias occurred whereby individuals who are less educated and do not exercise regularly are under-represented. This is also supported by the fact that 68% of participants have the highest German school-leaving qualification. Additionally, self-selection bias towards physically active people as a result of the survey's subject matter may have occurred. Due to the relatively time-consuming design of the questionnaire, 12.7% did not complete the survey, possibly introducing further bias. Additionally, the implementation of self-report instruments in our study poses potential for social desirability and recall biases. The use of self-report alone to classify participants into the mentally healthy group may have introduced diagnostic uncertainty. Expert interviews may be a future way to address this issue in smaller follow-up studies. Finally, the cross-sectional design of our study does not allow us to draw conclusions about the causality of observed relationships.

## 5 Conclusion

Based on our findings, we recommend that more low-threshold public exercise opportunities (e.g., public fitness facilities) should be provided in public areas with few sports facilities and in childcare facilities. Low-cost exercise opportunities should also be created and promoted, especially for socially disadvantaged groups.

We further observed that the motives enjoyment, stress management, challenge, revitalisation, and affiliation play a crucial role for engaging in regular exercise. Therefore, it is essential to target and strengthen said motives in the general population.

Moreover, interventions to increase (exercise-related) self-efficacy could also be helpful in promoting regular exercise. Future studies should explore the direction of the relationship between personality traits and exercise, and how to best tailor exercise opportunities to the individual needs of exercise participants. We believe that addressing these suggestions in the future can make a significant contribution to promoting exercise, which in turn can significantly contribute to improving both physical and mental health in the population.

## Data Availability

The original contributions presented in the study are included in the article/Supplementary Material, further inquiries can be directed to the corresponding author.
