# Supplementary material for: Reasons to move—a cross-sectional study to identify factors promoting regular exercise
Source: Front Sports Act Living. 2024 Dec 11;6:1515687. doi: 10.3389/fspor.2024.1515687 (PMC11668576; doi:10.3389/fspor.2024.1515687)
Supplement: Supplementary file 1 [file Datasheet1.docx]

**Supplement – Reasons to move – a cross-sectional study to identify factors promoting regular exercise**

**S1 Reliability and validity information for the psychometric instruments used**

SSK scale

The scale for sport- and exercise-related self-concordance (SSK scale) was validated in two studies. The first study was conducted with students at the University of Freiburg, while the second involved randomly selected residents of the Freiburg urban area. A total of 669 individuals participated in the aforementioned studies. In addition to the instruments of the SSK questionnaire, two further measurement instruments were included in the study for the purpose of validation. A principal component analysis confirmed the instrument’s multidimensional structure for the measurement of sport- and exercise-related self-concordance (1).

EMI-2

The Exercise Motivations Inventory (EMI-2) was developed through open-ended questioning, in which individuals were asked to provide reasons for their engagement in physical exercise. The questionnaire was designed to test questions derived from theoretical models, with a particular focus on Deci and Ryan’s self-determination theory (SDT)(Ryan and Deci 2017). In different processes, the number of items was reduced from 71 to 44. The internal consistency of the items was evaluated using Cronbach’s alpha, and the test-retest reliability was assessed by administering the EMI once more to a group of 100 participants from the original sample four to five weeks after the initial administration. In further analyses the validity as a measure of individuals’ exercise motivations was supported by using the data gathered from 249 participants for the development of the questionnaire (2).

SSA scale

The SSA scale was developed with the objective of reliably measuring the construct of ‘self-efficacy for sporting activity’ in German-speaking countries using a questionnaire. In order to determine the scale’s validity, it was compared with other instruments. The results of the reliability analyses demonstrated good internal consistency and unidimensionality (3).

BFI-10

In order to ensure the psychometric quality of the Big-Five-Inventory-10 (BFI-10), two extensive samples comprising a total of 1,673 participants were collected. Sample 1 was a quota sample, while Sample 2 was a random sample. Validation was conducted using both established standard instruments and specially developed short scales. The surveys were conducted by independent commercial providers. To test the psychometric quality of the BFI-10, a series of key values were calculated in relation to reliability and a number of aspects of validity. The reliability of the BFI-10 scale was determined using a retest with an interval of six to eight weeks. All reliability coefficients fell within the range deemed sufficient for group studies. The internal structure was evaluated using a heterogeneous sample. In addition, various aspects of construct validity were analysed (4).

IE-4 scale

The IE-4 scale for internal and external locus of control is based on classical test theory. The scale was validated by two extensive samples with 1,673 participants in total. The first study population was a quota sample, stratified according to the characteristics of gender, age, education and federal state with the population defined as ‘all German-speaking persons aged 18 and over living in private households in the Federal Republic of Germany’. The survey was conducted in two waves with a time interval of six to ten weeks. The second study population was a random sample. In order to check the psychometric quality of the constructed scale, characteristic values for reliability and various aspects of validity were calculated on the basis of the two samples.

It has been documented that validation studies confirm a stable two-factor structure. The results observed largely corresponded to the findings reported in the literature on the construct of locus of control. The construct validity of the IE-4 could thus be proven. The reliability of the IE-4 is stated as sufficient for group studies (5).

ASKU

The global quality of the instrument for measuring general self-efficacy can be evaluated as satisfactory, which allows for the conclusion to be drawn that the scale has factorial validity. The instrument demonstrated sufficient levels of reliability for the purposes of a group study and the objectivity can be considered as given (6).

BRS

The German version of the Brief Resilience Scale was validated using data from a population-based and a representative sample with a total of 2,709 participants. The reliability and validity as well as the unidimensional structure of the scale were demonstrated. Reliability analysis was performed using Cronbach’s α with α = .85 in both samples, indicating good reliability (7).

R1

Construct validity of the instrument for measuring risk taking was ensured by closely matching the wording of the item to the definition of the construct. The results of the construct validity test were consistent with previous findings. The results of the reliability were also in line with previous findings (8).

**Table S2 English translation of the questionnaire used**

**~~~~**

**~~~~**

**~~~~**

**~~~~**

**~~~~**

**~~~~**

**~~~~**

**~~~~**

**~~~~**

**~~~~**

**~~~~**

**~~~~**

Table S2 shows the English translation of the questionnaire used. GPAQ, Global Physical Activity Questionnaire (9); SSKS, “Sport- und Bewegungsbezogene Selbskonkordanz-Skala” (surveys different motives for exercising)(10); EMI-2, Exercise Motivations Inventory (11); SSA, Self-efficacy for sporting activity (3); BFI-10, Big Five Inventory 10 (4); IE-4, Internal-External Control Belief Scale (5); ASKU, General Self-Efficacy Short Scale (6); BRS, Brief Resilience Scale (7); R1, Brief Scale for Risk Taking (8).

**Table S3 Demographic characteristics**

Table S3 shows descriptive statistics for demographic characteristics for the general population sample. Absolute numbers in brackets (); SD, standard deviation

**Table S4 Summary of the EMI-2 results**

Table S4 presents descriptive and t-test statistics for the significant extrinsic and intrinsic motives of the EMI questionnaire, comparing the exercising sample with the non-exercising sample of the mentally healthy participants. EMI, Exercise Motivations Inventory (11); M, mean; SD, standard deviation; p, p value; df, degrees of freedom; T, t value; MD, mean difference; CI, confidence interval.

**References**

1. Seelig H, Fuchs R. Messung Der Sport- Und Bewegungsbezogenen Selbstkonkordanz. *Zeitschrift für Sportpsychologie* (2006) 13 (4), 121-139.

2. Markland D, Hardy L. The Exercise Motivations Inventory: Preliminary Development and Validity of a Measure of Individuals' Reasons for Participation in Regular Physical Exercise. *Personality and Individual Differences* (1993) 15(3):289-96. doi: 10.1016/0191-8869(93)90219-S.

3. Fuchs R, Schwarzer R. Selbstwirksamkeit Zur Sportlichen Aktivität: Reliabilität Und Validität Eines Neuen Meßinstruments - Self-Efficacy Towards Physical Exercise: Reliability and Validity of a New Instrument. *Zeitschrift für Differentielle und Diagnostische Psychologie* (1994) 15:141-54.

4. Rammstedt B, Kemper CJ, Klein MC, Beierlein C, Kovaleva A. *Eine Kurze Skala Zur Messung Der Fünf Dimensionen Der Persönlichkeit: Big-Five-Inventory-10 (Bfi-10)*. In: Sozialwissenschaften G-L-If, editor. GESIS-Working Papers. Köln, Germany: GESIS - Leibniz-Institut für Sozialwissenschaften (2012). p. 30.

5. Kovaleva A, Beierlein C, Kemper CJ, Rammstedt B. *Internale-Externale-Kontrollüberzeugung-4 (Ie-4)*. Köln, Germany: GESIS - Leibniz-Institut für Sozialwissenschaften (2014).doi: 10.6102/zis184.

6. Beierlein C, Kovaleva A, Kemper CJ, Rammstedt B. Allgemeine Selbstwirksamkeit Kurzskala (Asku). *Zusammenstellung sozialwissenschaftlicher Items und Skalen (ZIS)* (2014). doi: 10.6102/zis35.

7. Chmitorz A, Wenzel M, Stieglitz RD, Kunzler A, Bagusat C, Helmreich I, et al. Population-Based Validation of a German Version of the Brief Resilience Scale. *PLoS One* (2018) 13(2):e0192761. Epub 20180213. doi: 10.1371/journal.pone.0192761.

8. Beierlein C, Kovaleva A, Kemper CJ, Rammstedt B. Kurzskala Zur Erfassung Der Risikobereitschaft (R-1). *Zusammenstellung sozialwissenschaftlicher Items und Skalen (ZIS)* (2015). doi: 10.6102/zis236.

9. WHO. *Global Physical Activity Questionnaire (Gpaq)*. In: Department PoND, editor. Geneva, Switzerland: World Health Organization (2021).

10. Seelig H, Fuchs R. Messung Der Sport- Und Bewegungsbezogenen Selbstkonkordanz. *Zeitschrift für Sportpsychologie* (2006) 13 (4):121-39.

11. Markland D. *The Exercise Motivations Inventory - 2 (Emi-2)*. In: Bangor UK, editor.: Bangor University (1997).
